# Adjustment of Subthalamic Deep Brain Stimulation Parameters Improves Wheeze and Dyspnea in Parkinson's Disease

**DOI:** 10.3389/fneur.2019.01317

**Published:** 2019-12-13

**Authors:** Hiroyasu Komiya, Katsuo Kimura, Hitaru Kishida, Takashi Kawasaki, Koichi Hamada, Hiroyuki Koizumi, Naohisa Ueda, Fumiaki Tanaka

**Affiliations:** ^1^Department of Neurology, Yokohama City University Medical Center, Yokohama, Japan; ^2^Department of Neurosurgery, Yokohama City University Medical Center, Yokohama, Japan; ^3^Department of Neurology and Stroke Medicine, Yokohama City University Graduate School of Medicine, Yokohama, Japan

**Keywords:** deep brain stimulation, wheeze, Parkinson's disease, hyperadduction of the false vocal fold, laryngeal dystonia

## Abstract

Subthalamic nucleus deep brain stimulation (STN-DBS) is an effective treatment for motor features in Parkinson's disease (PD). We present the case of a 56-year-old man with a 17-year history of PD. He underwent bilateral STN-DBS at the age of 51 years because of troublesome dyskinesia and wearing off. His motor features dramatically improved after the operation; however, he developed dysarthria and a refractory wheeze associated with dyspnea due to abnormal hyperadduction of the false vocal fold. By adjusting the stimulation site of STN, his severe wheeze, which was considered to be the result of the unfavorable spread of current to the corticobulbar tract, was significantly improved. This report provides concrete evidence that wheezing is caused by hyperadduction of the false vocal fold as an adverse effect of STN-DBS and can be reversed by adjusting the stimulation site for STN-DBS.

## Background

Subthalamic nucleus deep brain stimulation (STN-DBS) is an effective treatment for motor features in Parkinson's disease (PD). However, stimulation of adjacent structures such as the corticobulbar tract (1–3) or cerebellothalamic tract (4, 5), may develop unwanted side effects, including speech deterioration and paresthesia. In this regard, electrode position is one of the determinant factors for stimulation outcome. Wheeze, or dyspnea, has been reported as a rare adverse effect of STN-DBS in a few previous studies (6, 7) but its underlying mechanisms have not yet been fully elucidated. Moreover, defining a treatment strategy for controlling the stimulation parameters remains challenging. Here, we report a case of PD in which a severe wheeze and dyspnea caused by hyperadduction of the vocal cords, an unreported mechanism for dyspnea induced by STN-DBS, were improved by adjustment of the stimulation parameter (i.e., electrode contact site).

## Introduction

**Figure 1:**
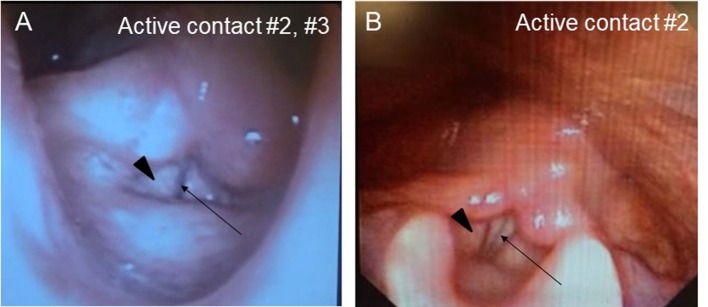
Nasopharyngeal endoscopy view represents vocal fold (arrow) and false vocal fold (arrowhead). **(A)** Hyperadduction of the false vocal fold in the setting of active contact at sites #2 and #3. **(B)** The hyperadduction of the false vocal fold was improved by changing the active contact to site #2.

**Figure 2:**
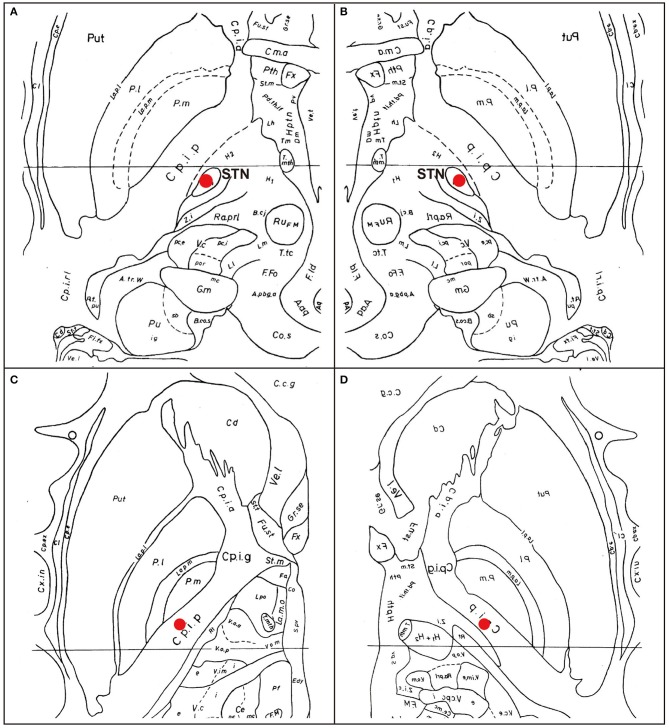
Eelectrode contact positions. The analysis of the electrode contact positions, mapping on the Schaltenbrand and Wahren (8). **(A)** The location of the contact #2 on the right electrode. The marker indicates the bottom of the contact #2, located at the dorso-lateral border of the right STN. **(B)** The location of the contact #2 on the left electrode. The marker indicates the center of the contact #2. **(C)** The location of the contact #3 on the right electrode. The marker indicates the center of the contact #3, located at the internal capsule. **(D)** The location of the contact #3 on the left. The marker indicates the center of the contact #3, located at the internal capsule. Put, putamen; Cd, caudate nucleus; STN, subthalamic nucleus; Pl, external segment of globus pallidus; Pi, internal segment of globus pallidus; Cpia, anterior limb of internal capsule; Cpip; anterior limb of internal capsule; Zi, zona incerta; Ru, red nucleus.

A Japanese man with a history of mild asthma was diagnosed with PD at the age of 39 years because of the presence of cogwheel rigidity and bradykinesia in his right upper and lower limbs. His motor features were effectively controlled by oral medications during the initial few years; however, at the age of 43, he developed troublesome dyskinesia and wearing off, which gradually became difficult to control. Accordingly, he underwent bilateral STN-DBS using model 3387 electrodes with Soletora^®^ implantable pulse generators (Medtronic, Minneapolis, MN, USA) at the age of 51 years, and his motor features dramatically improved. However, he developed dysarthria, spastic gait disturbance, and a refractory wheeze associated with dyspnea resistant to anti-asthmatic agents, which gradually deteriorated over several years. At the age of 56 years, he was admitted to our hospital in an attempt to adjust the DBS settings. At that time, he was taking levodopa/carbidopa 200 mg/day and ropinirole 8 mg/day. At the time of admission, his Movement Disorder Society Unified Parkinson's Disease Rating Scale (MDS-UPDRS) part III and part IV scores were 40 and 1, respectively in the on-medication and on stimulation state. Nasopharyngeal endoscopy showed hyperadduction of the vocal cords, accounting for his wheeze (Figure 1A). The following stimulation conditions were used: voltage of 3.2 V, pulse width of 90 μs, and frequency of 185 Hz per second. Relatively high values of these parameters were selected in the previous hospital presumably to obtain enough DBS effects for improving dyskinesia and wearing off. Therefore, we reduced the stimulation voltage, pulse width, and/or frequency, resulting in mild attenuation of the adverse effects, but troublesome dyskinesia and wearing off reappeared. In order to confirm the precise location for the STN-DBS electrodes, SurgePlan^®^ system (ELEKTA Neuroscience, Stockholm, Sweden) was employed with reference to the brain anatomic images taken by 1.5-Tesla 3-dimensional spoiled gradient magnetic resonance imaging and computed tomography. Each contact site detected by this analysis is described in Table 1, Figure 2. An implantable pulse generator was set as the anode and the cathode was set at contact sites #2 and #3 on admission. Because the contact site #3 on both sides was located near the internal capsules, it was considered that the excess muscle contractions caused by stimulation of the motor fibers by the extra spread current may have led to the hyperadduction of the vocal cords, dysarthria, and spastic gait disturbance. Accordingly, preserving above stimulation conditions, his DBS cathodes active at contact sites #2 and #3 were changed to be active at contact site #2 (Table 1), resulting in significant and immediate improvements in the severe wheeze, dyspnea (Video S1), speech impairment, and walking disability. The MDS-UPDRS part III and part IV scores were also improved to 29 and 0, respectively. The normalization of the false vocal fold constriction was confirmed by nasopharyngeal endoscopy (Figure 1B, Video S2). His dyskinesia and wearing off were well controlled.

**Table 1:** Parameters of STN-DBS in the patient.

	**Left hemisphere**	**Right hemisphere**
**Location of the contact**	**Medtronic #3387 electrode**	
Contact #0	SN	SN
Contact #1	STN	STN
Contact #2	STN/ZI	STN/ZI
Contact #3	IC/Th	IC/Th
**Setting at admission**		
Stimulation	Monopolar	Monopolar
Cathode	#2, #3	#2, #3
Anode	IPG	IPG
Voltage	3.2 V	3.2 V
Pulse width	90 μs	90 μs
Frequency	185 Hz	185 Hz
**Setting changed**		
Stimulation	Monopolar	Monopolar
Cathode	#2	#2
Anode	IPG	IPG
Voltage	3.2 V	3.2 V
Pulse width	90 μs	90 μs
Frequency	185 Hz	185 Hz

*SN, substantia nigra; STN, subthalamic nucleus; ZI, zona incerta; IC, internal capsule; Th, lateral thalamus; IPG, implantable pulse generator; msec., microseconds; Hz, hertz (pulse per second)*.

## Discussion

Speech disorder with variable presentations, such as weak voice, monotony of pitch and loudness, short rush of speech and dysarthria, is observed in the majority of patients with PD (4). Although pharmacological therapies are generally beneficial for the motor features, they are not always effective for the parkinsonian speech (9). In addition, although STN-DBS is also an effective treatment for motor features in PD, it is still controversial whether such neurosurgical procedures have favorable (10) or unfavorable effects (1, 4) on the speech in patients with PD. In our case, dysarthria emerged as an unfavorable effect of STN-DBS. Additionally, our patient developed a refractory wheeze after STN-DBS. The wheeze in our case was induced by abnormal hyperadduction of the false vocal fold as revealed by the endoscopic examination. Interestingly, a significantly higher incidence of abnormal hyperadduction of the false vocal fold without wheeze or dyspnea has been reported in patients who received STN-DBS compared to those without DBS treatment (11). In our case, adverse effects of STN-DBS including dysarthria, spastic gait disturbance, and a refractory wheeze were enhanced immediately after stimulation was increased and attenuated by decreased stimulation, which is characteristic of capsular effects on the pyramidal tract causing paresis and increased muscle tone. Moreover, as shown in Figure 2, the bilateral cathode contact (contact #3) was located at very close proximity to the internal capsule, which prompted us to consider that the dysarthria was caused by the extra current spread to the corticobulbar tract. In our case, modifying the stimulation parameters, such as reducing voltage, attenuated side effects by preventing the extra current spread, but also diminished the beneficial effects on his rigidity, bradykinesia, dyskinesia, and wearing off. Notably, switching the active contact from site #2 and #3 to site #2, without changing the other stimulation parameters including voltage, pulse width, and stimulation frequency (voltage of 3.2 V, pulse width of 90 μs, and frequency of 185 pulse per second), improved not only the dysarthria, but also the wheeze and the false vocal fold hyperadduction, preserving the beneficial effects on his rigidity, bradykinesia, dyskinesia, and wearing off. Moreover, spastic gait disturbance was also improved. Our contact #3 was somatotopically localized at or near the leg region of the corticospinal tract (Figure 2), which is in agreement with the capsular effect on the leg and spreading effect on the face (including vocal cord) region of the corticobulbar tract located near the genu of internal capsule. Another explanation for the abnormal hyperadduction of the false vocal cord might be extrapyramidal mechanisms causing dystonia due to inhibition of pallido-thalamo-cortical interconnections (12). The mechanism of previously reported STN-DBS-induced fixed epiglottis associated with dyspnea was attributed to dystonia induced by high voltage stimulation, although the position of the simulating area was not discussed (6). In our case, considering the relative positional relationship of neuroanatomical structures shown in Figure 2 and lack of coexistence of dystonic features in the other body parts, we favor capsular effects rather than dystonia as an explanation for the abnormal hyperadduction of the false vocal cord. Interestingly, when using the single monopolar #3, the wheeze did not improve, which indicates the importance of active contact sites. This finding is consistent with the efficacy of the adjustment of the active contact location recently illustrated by cases of improvement of speech intelligibility (11). On the other hand, no correlation was observed between dyspnea severity and stimulation parameters including electrode position in a prospective, longitudinal study using respiratory questionnaire that showed relatively higher incidence of dyspnea after STN-DBS compared to ventral intermediate thalamus DBS (7). As the authors suggested, the STN itself may play a role in respiratory perception or control and its stimulation may cause mild dyspnea detectable by questionnaire. However, severe and relatively acute dyspnea, as in our case, is caused by extra spread current to the surrounding structures, such as internal capsule, but not by the STN stimulation itself, which also accounts for the improvement by modulation of electrode contact position.

In conclusion, the immediate amelioration of the wheeze by the active contact modulation in our case strongly suggests that the wheeze was the result of unfavorable spread of current to the corticobulbar tract. Thus, clinical attention should be paid not only to speech deterioration but also to a wheeze caused by a current spread to inappropriate regions, such as the internal capsule, in bilateral STN-DBS. These adverse effects can be effectively reversed and the activities of daily living can be well-maintained by adjusting the stimulation site for STN-DBS using minute image assessment.

## Ethics Statement

Written informed consent was obtained from the individual(s) for the publication of any potentially identifiable images or data included in this article.

## Author Contributions

HKom, TK, HKis, HKoi, KH, and KK: examination, diagnosis, therapy of the patient, and drafting the manuscript. NU and FT: study design, supervision of data analysis, interpretation, evaluation of clinical data, and drafting the manuscript.

### Conflict of Interest

The authors declare that the research was conducted in the absence of any commercial or financial relationships that could be construed as a potential conflict of interest.

## Supplementary Material

Video S1 Severe wheeze by STN-DBS and improvement of the wheeze after changing the DBS contact site are shown in the first half and the latter half of the movie, respectively.

Video S2 Nasopharyngeal endoscopy view represents vocal fold and false vocal fold. The first half of the movie shows the hyperadduction of the false vocal fold induced by STN-DBS. In the latter half of the movie, the normalized contraction of the false vocal fold after changing the DBS contact site is presented.
